# Effect of Corn Starch Granules on Stabilizing the Foam Structure of Ultrasonically Modified Whey Isolate Protein

**DOI:** 10.3390/foods11223572

**Published:** 2022-11-10

**Authors:** Jingxue Liu, Jiaying Xin, Tingting Gao, Guiru Wang, Chungu Xia

**Affiliations:** 1 Key Laboratory for Food Science and Engineering, Harbin University of Commerce, Harbin 150028, China; 2 College of Food Engineering, Jilin Agricultural Science and Technology University, Jilin 132101, China; 3 State Key Laboratory for Oxo Synthesis & Selective Oxidation, Lanzhou Institute of Chemical Physics, Chinese Academy of Sciences, Lanzhou 730000, China

**Keywords:** whey protein isolate, corn starch granules, ultrasound, foam properties, molecular structure, intermolecular interaction

## Abstract

In this study, the mechanism of ultrasound combined with corn starch granules (CSG) treatment improved the foam properties of whey protein isolates (WPI) and was systematically investigated. The results showed that ultrasound combined with corn starch granules treatment increased foam capacity and stability by 15.38% and 41.40%, respectively. Compared with the control group, corn starch granules enhanced the surface charge (52.38%) and system turbidity (51.43%), which certainly provided the necessary conditions for the improvement of foam stabilization stability. In addition, corn starch granules as microgel particles increased the mechanical properties of the interfacial protein film, thus delaying the instability of foam. This research would provide new insights into the design of new protein-based foam foods in the future food industry.

## 1. Introduction

Whey protein isolate (WPI) is mainly composed of β-lactoglobulin (50%), α-lactalbumin (20%), bovine serum protein (4.7%), and immunoglobulin (3%) [1]. The rigid structure in its spatial conformation is considered to be the basis for its application in food processing [2]. In general, food proteins can act as good foaming agents to give foam-based foods a satisfying appearance, taste, and texture, such as ice cream, angel cake, and salad dressings [3]. However, under the diverse environmental conditions of food processing, thermally unstable protein foam systems are not very stable [4]. Numerous factors will adversely affect the foam capacity and stability of protein solutions.

In response to this situation, researchers have attempted to improve protein foaming systems using a variety of methods [2,5,6]. Ultrasound processing, a non-additive and non-thermal physical processing method, has aroused the strong concern among scholars [7]. Proteins treated by moderate ultrasound tend to exhibit good interfacial activity, which is mainly attributed to the cavitation effect, thermal effect, and local high pressure caused by ultrasound [8,9]. With these effects, the hydrophobic groups inside the protein are exposed, enhancing its interfacial adsorption capacity. It has been demonstrated that ultrasonically modified proteins will be adsorbed more rapidly at the air-water interface under mechanical stirring, so as to give the protein system a more remarkable foam capacity [10]. It is indeed a promising processing technology in the food industry.

Although the foam capacity is enhanced, the formed foam system tends to destabilize within a short period of time due to the fact that the interfacial film structure formed by proteins at the air-water interface is still relatively weak. This phenomenon is likewise a factor that cannot be ignored in restricting the development of protein foam-based foods. Therefore, a simple and efficient foam stabilization technique may be beneficial to solve this problem. Recently, several teams have found that microparticles formed from biomacromolecules might possess excellent interfacial stabilization [3,11]. Protein microgels and starch granules have been shown to be embedded at the air-water interface under specific conditions to achieve stability of the foam system [3,11]. These microparticles are stabilized at the interface similar to armor, which on the one hand prevents gas exchange on both sides of the interface and on the other hand inhibits the proximity and agglomeration of adjacent bubbles [12]. In addition, starch-induced protein structural transformation is also crucial for the stability of protein foam. Due to the addition of starch granules, the spatial arrangement of the original hydrophilic and hydrophobic domains in the protein structure may be changed, which will theoretically affect the interfacial film formation behavior of proteins. In other words, starch particles mainly achieve foam stability by affecting the mechanical strength of the interface film and the protein structure.

Therefore, we considered whether corn starch granules (CSG), a common food matrix, could solve the problem of unstable WPI foam. In this work, ultrasonically modified WPI was first prepared to confirm its improved effect on foam capacity. In addition, bulk phase and molecular characteristics were evaluated to discuss the mechanism of ultrasound and corn starch granules affecting the properties of WPI foam.

## 2. Materials and Methods

### 2.1. Materials

WPI and corn starch granules were purchased from Sinopharm Chemical Reagent Co., Ltd. (Shanghai, China) Trypsin and ANS were obtained from Sigma company. Other necessary reagents (analytically pure) were provided by Shanghai yuanye Bio-Technology Co., Ltd. (Shanghai, China).

### 2.2. Preparation of Ultrasonically Modified WPI

The WPI was carefully dissolved in deionized water and its concentration was set to 20 mg/mL. HCl and NaCl solutions (0.1 mol/L) were used to adjust the pH of the protein solution to 7.0. Next, the prepared protein solutions (300 mL) were subjected to ultrasound modification (SCIENTZ-IID, NingBo Scientz Biotechnology Co. Ltd., Ningbo, China). The ultrasound generator probe (10 mm) was placed 5 cm below the solution level and the entire vessel was placed in an ice water bath to avoid overheating. The samples were treated at 120 W (10.63 W/cm^2^), 240 W (21.26 W/cm^2^), and 360 W (31.89 W/cm^2^) for 30 min, respectively, with a 5 s pulse interval [13]. Thermocouple sensors were employed to monitor the sample temperature in real-time and control the sample temperature to 25 °C by adjusting the ice water bath temperature. WPI solution without sonication served as a control.

### 2.3. Development of Systems Containing Corn Starch Granules

While the WPI solution was prepared, cornstarch granules were dissolved in deionized water to make a solution with a concentration of 20 mg/mL. Then, the protein solution and starch solution were mixed at a ratio of 1:1 (*v*/*v*) and stirred (100 r/min) for 2 h to ensure adequate hydration. The prepared mixed systems were treated according to the same ultrasound strategy.

### 2.4. Measurement of Foam Properties

The sample solutions were carefully transferred into graduated measuring cylinders. A high-speed disperser was applied to stir at 8000 r/min for 60 s. After foaming, the foam was allowed to stand at room temperature for 30 min to test the foam stability. Foam capacity and stability were calculated according to the following equations [2].

$$\mathrm{Foam}\;\mathrm{capacity}=\left(V_{2}-V_{1}\right)∕V_{1}\times 100$$

$$\mathrm{Foam}\;\mathrm{stability}=\left(V_{3}-V_{1}\right)∕V_{1}\times 100$$

where V_1_, V_2_, and V_3_ represent the initial volume, the volume just after stirring, and the volume after 30 min, respectively.

In addition, after 30 min of standing, photographs of the bubble morphology were collected using an optical microscope, and the average diameter of the bubbles was further analyzed using image J software.

### 2.5. Turbidity Measurement

The absorbance (A) of the sample solution at 400 nm was measured at 25 °C. The transmittance (T) of each sample was further calculated according to the Lambert-Bier law (A = −lgT). Turbidity was then defined according to the following equation [14]: Turbidity (%) = 100 − T (%).

### 2.6. Zeta Potential Measurement

The protein concentration in the samples was adjusted to 1 mg/mL with deionized water. Zeta potential was measured using a Zetasizer Nano ZS (Malvern Instruments, Southborough, UK) at 25 °C [15].

### 2.7. Measurement of Surface Hydrophobicity

The protein concentration of each sample was diluted to 0.1-0.5 mg/mL. These samples (2 mL) and the ANS solution (10 μL, 8 mmol/L) were then mixed well and left to stand for 30 min protected from light. The IF-5401 spectrofluorimeter (Hitachi, Japan) was utilized to detect the fluorescence spectrum of each sample. The excitation wavelength, emission wavelength, and slit width were 390 nm, 470 nm, and 5 nm, respectively [16]. The test temperature was 25 °C. Surface hydrophobicity was defined as the initial slope of the fluorescence intensity-protein concentration curve.

### 2.8. Molecular Flexibility Measurement

The protein concentration of the samples was adjusted to 1 mg/mL. Trypsin was dissolved in Tris-HCl buffer (pH 7.2, 5 mmol/L) to a concentration of 1 mg/mL. Subsequently, the samples and the trypsin solution were well mixed and placed in a 38 °C water bath for 5 min. Trichloroacetic acid (4 mL, 5%) was added to the mixture to terminate the enzymatic reaction. After the enzymatic digestion product was centrifuged (4000× *g*, 30 min, 4 °C), the supernatant was carefully removed and the absorbance was measured at 280 nm. This absorbance was defined as the molecular flexibility of the protein [12].

### 2.9. Circular Dichroism Measurement

The samples whose protein concentration was diluted to 0.5 mg/mL were detected by the far-UV spectrum with a Jasco-810CD spectropolarimeter (Jasco, Japan). The wavelength range was 190–250 nm with a resolution of 0.2 nm [17]. The scan speed, response time, and bandwidth were 100 nm/min, 0.25 s, and 1.0 nm, respectively. The exact secondary structure content was calculated by fitting the spectra to Young’s equation.

### 2.10. Intermolecular Force Measurement

First, five solutions [A (0.05 mol/L NaCl), B (0.6 mol/L NaCl), C (0.6 mol/L NaCl and 1.5 mol/L urea), D (0.6 mol/L NaCl and 8 mol/L urea), and E (0.6 mol/L NaCl, 8 mol/L urea and 0.05 mol/L beta-mercaptoethanol)] were configured. Each sample solution was mixed with each of the above five solutions and fully reacted (4 °C, 1 h). The reacted samples were centrifuged at 10,733× *g* for 15 min. The absorbance of the supernatant at 280 nm and 260 nm was obtained to calculate the protein content. The differences between A and B, B and C, C and D, and D and E were defined as ionic bonding, hydrogen bonding, hydrophobic interactions, and disulfide bonding content, respectively.

### 2.11. Data Analysis

All tests were performed in triplicate. SPSS 25 and Origin 8.0 were employed for the statistical and visualization of the data. Significance analysis was performed at the 5% level.

## 3. Results and Discussion

### 3.1. Foam Properties Analysis

**Figure 1:**
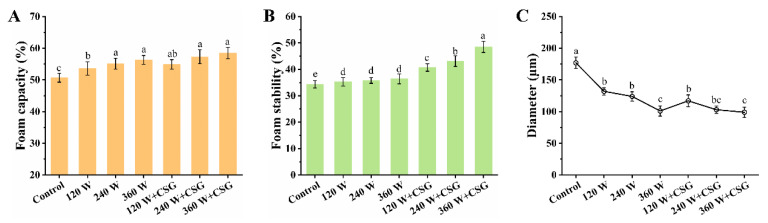
Foam capacity (**A**), foam stability (**B**), and average foam diameter (**C**) of WPI and WPI–CSG systems treated by different power ultrasounds. Different lowercase letters represent significant differences (*p* < 0.05) between data.

In Figure 1, the foam characteristics of the modified WPI were evaluated comprehensively in terms of foam capacity, foam stability, and average bubble diameter, respectively. As the trend presented in Figure 1A, ultrasound treatment significantly enhanced the foam capacity of the samples compared to the control group (*p* < 0.05). Meanwhile, corn starch granules did not seem to improve the protein foam capacity, as the assay results of the treatment group with added starch granules did not exhibit a significant enhancement at the same ultrasound power (*p* > 0.05). This phenomenon might be attributed to the following aspects. On the one hand, ultrasound treatment did induce structural changes in WPI, which enhanced the interfacial activity of proteins to a certain extent and accelerated their diffusion and adsorption to the air-water interface [18]. This mechanism has been demonstrated in previous studies on ultrasound modification of food proteins [10]. The cavitation and thermal effects induced by moderate intensity ultrasound provide favorable conditions for the unfolding and rearrangement of protein structures. On the other hand, although previous studies have shown that starch has the potential to change protein conformation, it is not directly involved in the formation of interfacial membrane structures [19]. Therefore, the transformation of protein spatial structure due to corn starch granules did not seem to significantly enhance the foam capacity of WPI.

Even so, the foam stability data in Figure 1B illustrated the good ability of corn starch granules in stabilizing the foam structure of WPI. As shown in Figure 1B, while ultrasound significantly improved foam stability (*p* < 0.05), corn starch granules further enhanced this effect, which continued to increase with increasing ultrasound intensity. In particular, the corn starch granules increased the foam stability to a maximum of 48.5% under ultrasound treatment at 360 W, which was a 33.2% improvement over ultrasound treatment alone. Recently, many scholars have proposed that starch granules tend to bind to proteins in a macromolecular complex, and this complex stabilizes the interface by adsorbing on the side of the air-water interface close to the aqueous phase [15,19]. Similarly, microgel particles formed by proteins under moderate thermal induction also possess the ability to stabilize foam systems in this way. While altering the secondary structure of WPI, the physical barrier formed by starch granules at the interface also plays an indispensable role in the stabilization of the foam structure.

Additionally, in Figure 1C, the average diameter of the bubbles after 30 min decreased continuously as the ultrasound treatment power increased. In general, smaller bubble sizes in the foam system tend to suggest a more stable overall structure [12]. The dense and uniform small bubbles were close to each other to form a reliable and firm Plateau interface, which will undoubtedly enhance the stability of the foam system. The fact that ultrasound contributed to improving the structural stability of protein-based foams was once again confirmed. However, no significant effect of corn starch granules on bubble size was observed (*p* > 0.05), which might be due to that starch granules were located in the aqueous phase, making it difficult to directly affect the bubble morphology significantly. In addition, those relatively unstable bubbles had all disappeared during the stabilization of the system. The remaining bubbles were relatively stable in diameter, which tended to indicate lower interfacial tension. Generally, low interfacial tension corresponded to high interfacial protein adsorption, indicating that starch particles had no obvious impact on the amount of adsorbed protein at the interface when the system was stable. In conclusion, even though the foam capacity and bubble size were not observed to be favorably altered compared to the samples treated with ultrasound only, the foam stability of WPI with the addition of corn starch granules was greatly enhanced. The mechanism that underlies these phenomena is worthy of further discussion.

### 3.2. Turbidity Analysis

**Figure 2:**
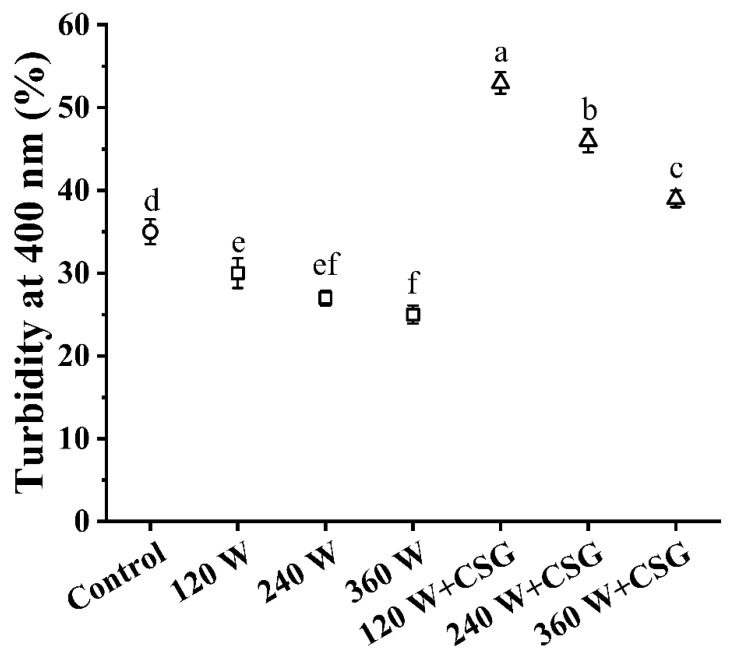
Bulk phase turbidity of WPI and WPI–CSG systems treated by different power ultrasound. Different lowercase letters represent significant differences (*p* < 0.05) between data.

Turbidity, an important indicator of the dispersion state and hydration properties of biomacromolecules in the bulk phase, has been widely utilized to evaluate the foaming potential of food matrices. The turbidity of the bulk phase for each treatment group was shown in Figure 2. It is obvious that the ultrasound treatment significantly reduced the turbidity of the samples (*p* < 0.05) and the turbidity decreased continuously with increasing power. This was mainly caused by the external energy input brought by ultrasound. The larger biological macromolecules originally dispersed in the aqueous phase responded strongly to the light signal, but with the intervention of ultrasound, the initial equilibrium state of these aggregates was disrupted and caused an increased entropic effect on the system [2]. The large number of small molecules formed in this process and their more disordered dispersion state led to the stronger light transmittance of the system. In addition, the cavitation effect also caused changes in the structure of WPI during sonication. Their dense ordered molecular structure might become more spreading during vibration and accompanied by the outward migration of hydrophilic groups, and this speculation was confirmed by the data in Section 3.4 and Section 3.5. Furthermore, the turbidity of the samples with the addition of corn starch granules also decreased with increasing ultrasound power but was significantly higher overall than that of the samples treated with only ultrasound (*p* < 0.05). Such a phenomenon was consistent with our previous experience. Normally, starch-containing systems have a higher viscosity [20], which contributes to the stability of the colloidal system (Figure 1B). The high viscosity of the bulk phase inhibited foam agglomeration and disproportionation in the storage phase, which was a favorable foam stabilizer. At the same time, corn starch granules might block light transmission and increase system viscosity after water absorption and swelling. It has also been proposed that starch granules could act as Pickering particles to stabilize at the colloidal interface and form a physical barrier at the interface to inhibit protein desorption through a specific contact angle [21]. In conclusion, the results of turbidity analysis illustrated the fact that the ultrasound treatment altered the dispersion state of WPI in the aqueous phase and that corn starch granules might enhance the stability of proteins at the air-water interface.

### 3.3. Zeta Potential and Surface Hydrophobicity Analysis

**Figure 3:**
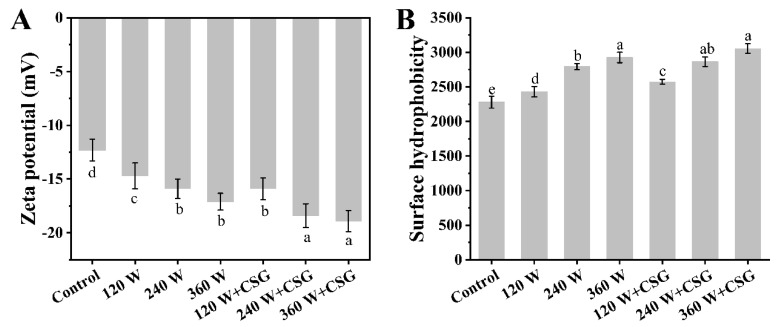
The zeta potential (**A**) and surface hydrophobicity (**B**) of WPI and WPI–CSG systems treated by different power ultrasound. Different lowercase letters represent significant differences (*p* < 0.05) between data.

Zeta potential is often employed to evaluate the dispersion state and particle morphology of colloidal particles in the bulk phase, which is a classical index to describe the surface properties of charged particles. Generally, colloidal particles at the interface exhibit a more spacious and disordered spatial conformation to maintain interfacial stability, and this state is dependent on a higher net surface charge [22]. The large net charge not only means that the amphiphilic molecules themselves can be embedded more efficiently on both sides of the interface but also helps to inhibit the proximity and aggregation of molecules to each other, which is essential for the formation of a homogeneous biomacromolecular network at the interface [23]. It could be clearly seen from Figure 3A that the surface charge of the WPI treated by ultrasound is significantly higher than that of the control group (*p* < 0.05). This alteration indicated that the ultrasound greatly affected the arrangement state of charged amino acid residues in WPI, and the protein surface electronegativity was considerably enhanced. Similar results have been obtained in other studies on ultrasonically modified proteins [18]. These large surface net charges ensured that the proteins were very evenly dispersed in the aqueous phase and could migrate rapidly to the air-water interface during agitation. Meanwhile, high electronegativity helped the interfacial protein membrane to maintain a dense and uniform state. More importantly, it could also be found from Figure 3A that the addition of corn starch granules significantly increased the net surface charge of the same sonicated protein (*p* < 0.05), and the zeta potential of the starch granules combined with 240 W and 360 W sonicated samples reached −18.4 mV and −18.9 mV, respectively. Theoretically, starch and proteins in the aqueous phase tend to bind at specific segments and side chains in the molecular structure through non-covalent interactions [24]. Among these non-covalent interactions, hydrogen bonding and electrostatic interactions were considered to be an extremely critical forces. A new balance of positive and negative charges inside the polymer was induced by them and further influenced the surface charge distribution. Hence, cornstarch granules might be an effective substance for improving the mechanical properties of interfacial macromolecular films [24].

In addition, the surface hydrophobicity of the samples with different treatments was given in Figure 3B. Surface hydrophobicity is an indispensable parameter for measuring the surface polarity of proteins. Since the affinity for proteins differs greatly on both sides of the air-water interface, surface hydrophobicity can be utilized to predict the probability of protein adsorption and stabilization at the interface [25]. As shown in Figure 3B, the surface hydrophobicity kept increasing significantly (*p* < 0.05) with the enhancement of ultrasound intensity. This phenomenon implied that the hydrophobic amino acid residues originally buried inside the protein molecule might be exposed to the molecular surface under the effect of ultrasound, which also suggested that the amino acid residue arrangement of the ultrasound-treated protein was strongly affected. Similar results have been previously observed in other researchers’ studies [26]. In this study, the ultrasound resulted in stronger intramolecular electrostatic repulsion as well as protein unfolding, thereby exposing hydrophobic amino acids. These extremely hydrophobic fragments were likely to be embedded on the side of the interface near the air during the evolution of the foam. What was surprising was that the corn starch granules did not seem to have a pretty intense effect on the surface hydrophobicity, even though a significant elevation was noticed at 120 W (*p* < 0.05). Starch granules were normally in the swollen state in the aqueous phase, and the increased surface area provided favorable conditions for the association between starch and protein. Nevertheless, since the surface hydrophobicity was not significantly enhanced at higher ultrasound powers (240 W and 360 W), it would be reasonable to hypothesize that the co-assembly between them did not involve hydrophobic fragments. On the whole, ultrasound contributed to improving the surface hydrophobicity and interfacial activity of WPI, but corn starch granules did not display an incredible effect in this respect.

### 3.4. Molecular Flexibility Analysis

**Figure 4:**
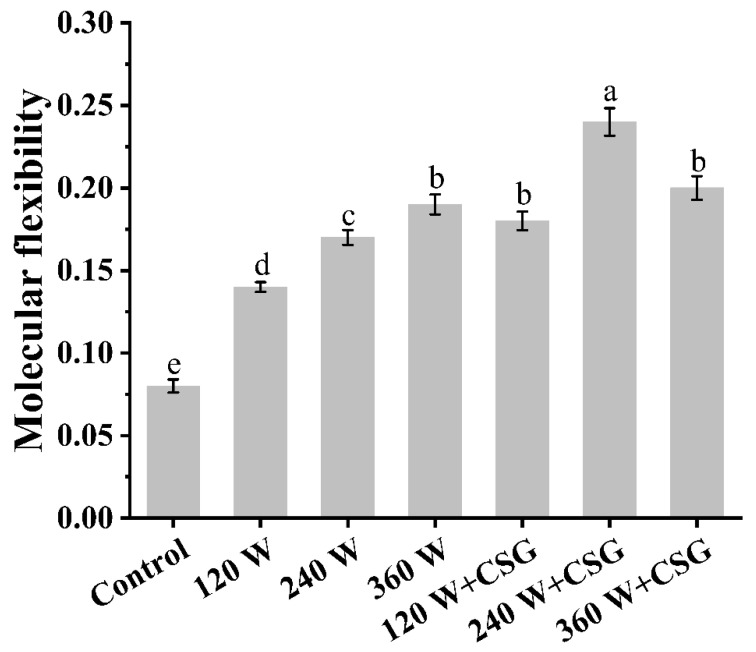
Protein molecular flexibility of WPI and WPI–CSG systems treated by different power ultrasound. Different lowercase letters represent significant differences (*p* < 0.05) between data.

Molecular flexibility has been widely recognized as one of the important factors determining the functional properties of proteins, which essentially reflects the motility of the protein molecular conformation. Furthermore, the interfacial properties of proteins, especially emulsification and foaming properties, are closely associated with the motility of the molecular conformation [27]. The transfer of proteins from the bulk phase to the interface generally undergoes three stages, namely diffusion, permeation, and rearrangement [12]. These processes are intensely dependent on suitable molecular flexibility. In Figure 4, both ultrasound treatment and the addition of corn starch granules significantly enhanced the molecular flexibility of WPI (*p* < 0.05), which reached a maximum value of 0.24 with the combined effect of 240 W ultrasound and starch granules. Some researchers have concluded that multiple forms of external energy could enhance the molecular flexibility of proteins [28,29]. During this transition, the ratio of the flexible to the rigid interval in the protein structure was moderately altered, which helped the molecules to permeate similar to hinges at the interface. In particular, the cavitation effect caused by ultrasound promoted the transformation of the rigid domain in the protein structure with strong local energy and made it adapt to the new interface force field environment [8]. Compared to the WPI treated with ultrasound only, the corn starch granules significantly improved the flexibility of the protein (*p* < 0.05), which could be confirmed in Figure 3. The starch granules induced further swelling of the protein structure to enhance its interfacial activity, and on the other hand gave a higher viscosity to the bulk phase, which would retard the foam destabilization by reducing the drainage rate [19]. As for why the molecular flexibility did not become higher as expected after the 360 W ultrasound treatment, the reason might be that the thermal effect caused by the excessive intensity of ultrasound caused the originally stretched protein molecules to regroup moderately. At this time, the protein skeleton structure was rigid, which inhibited the effective binding of trypsin to the active site to a certain extent. This phenomenon has also been observed in other studies on ultrasound-modified proteins [13]. Overall, the molecular flexibility of WPI was greatly enhanced after ultrasound and corn starch granules treatment, which was essential for the formation and stabilization of thermally unstable aqueous foams.

### 3.5. Secondary Structure Analysis

**Figure 5:**
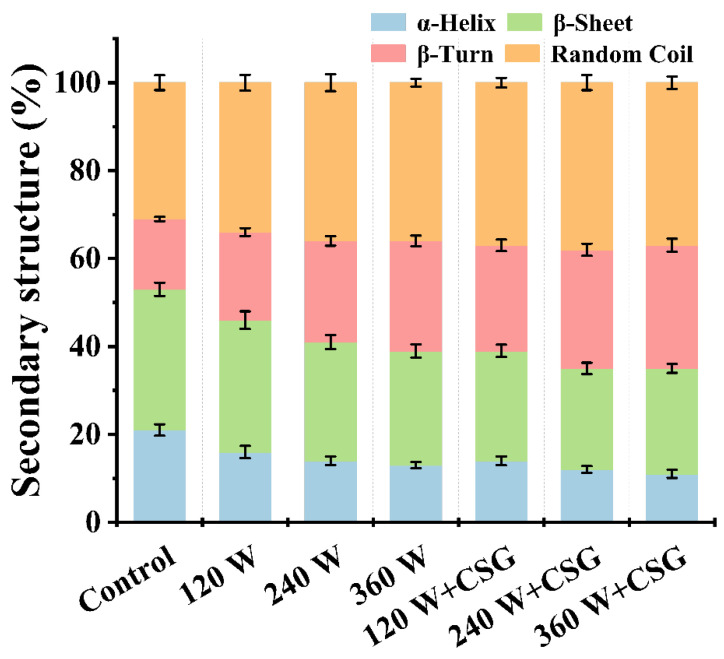
Protein secondary structures of WPI and WPI–CSG systems treated by different power ultrasound.

It has been clearly established that the molecular flexibility of WPI was significantly enhanced after treatment with ultrasound and corn starch granules, then further quantitative resolution of the protein secondary structure was indispensable. In comparison with the control group, ultrasound treatment increased the content of β-turn and random coil structures while reducing α-helix and β-sheet structures (Figure 5). It is known that the protein secondary structure relies mainly on intramolecular hydrogen bonds to maintain stability [30], so it can be inferred that the internal hydrogen bonds of WPI reached a new equilibrium state under the effect of ultrasound. The α-helix and β- sheet in the protein structure mainly undertake the role of maintaining a stable and ordered spatial conformation. Therefore, the structure of WPI might become more spreading and disordered with increasing ultrasound intensity, which was corroborated by the results of molecular flexibility analysis. This microstructural change provided the basis for the improvement of macroscopic foam properties, especially in terms of foam capacity (Figure 1A). Proteins with moderate structure stretching migrated rapidly to the vicinity of the interface under external perturbation. Subsequently, with the participation of both hydrophilic and hydrophobic groups, the entire molecule adsorbed to both sides of the interface in a sinuous manner and cross-linked into an interfacial mesh-like membrane structure within a short period of time [31]. Nevertheless, Figure 1A also demonstrated that the foam stability of the ultrasound-only treated samples was not remarkably enhanced. As well, this was mainly attributed to the fact that although these proteins would undergo structural rearrangement after adsorption, the overly flexible backbone was too weak to keep the rearranged membrane structure stable in the long term. Excitingly, the addition of corn starch granules has alleviated this situation. The results in Figure 1B illustrated that the corn starch granules successfully inhibited foam destabilization and improved the foam stability of WPI. The α-helix and β-sheet structure of the protein was further reduced after the addition of starch granules, which seemed to predict that the foam lifespan would not be too long. However, this was not the case. Starch granules have been proven to be a kind of Pickering particle with outstanding interfacial activity, which can be stably adsorbed on the air-water interface at a suitable contact angle for interfacial stabilization [32]. In this sense, the favorable effect of corn starch granules as interfacial stabilizers was much greater than the negative effect caused by the excessive stretching of the protein structure. Not only that, but the interactions between biological macromolecules in the system were also crucial for the impact of the evolutionary process of the foam.

### 3.6. Intermolecular Force Analysis

**Figure 6:**
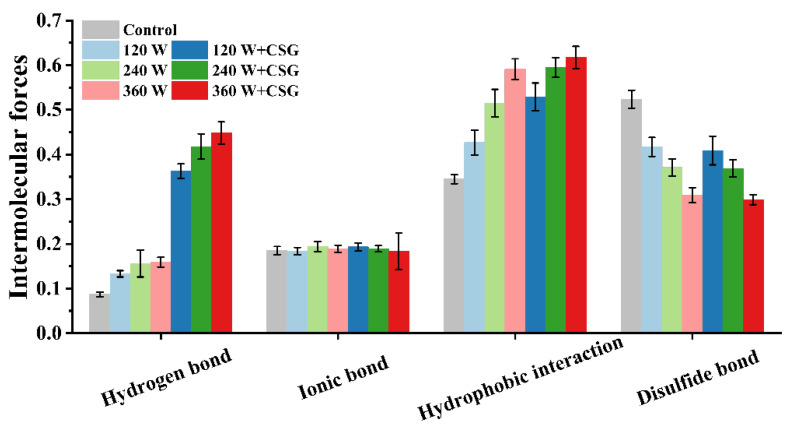
Intermolecular forces in WPI and WPI–CSG systems treated by different power ultrasound.

Intermolecular interactions in macromolecular systems are closely related to the macroscopic processing properties of complex food matrices. The changes in hydrogen bonding, ionic bonding, hydrophobic interactions, and disulfide bonding content of the systems subjected to different treatments were illustrated in Figure 6. For hydrogen bonding, the discussion above has pointed out that it is critical for maintaining the spatial conformation of proteins. The content of hydrogen bonds between WPI molecules increased slightly after ultrasound treatment, which was mainly caused by the change in protein conformation and dispersion state. After further ultrasound treatment of the system containing cornstarch granules, a substantial increase in hydrogen bonding was observed. The massive hydroxyl groups on the side chains of starch were not only responsible for increasing the viscosity of the bulk phase, but also induced non-covalent cross-linking between the starch granules and protein molecules [11]. In addition, interfacial water molecules possess the ability to form hydrogen bonds with proteins and starch, which will promote the long-term stability of the interfacial membrane structure. Ionic bonding, a type of salt bridge, normally refers to the electrostatic attraction between positive and negative ions [33]. In the system used in this study, the starch granules in the solution were generally considered uncharged, while the WPI was negatively charged. Therefore, the ionic bond content in the system did not change significantly regardless of the presence or absence of corn starch granules. The effect of ultrasound treatment on intermolecular interactions was also not reflected in the ionic bond content. Furthermore, the significant enhancement of hydrophobic interactions was consistent with the results in Figure 3B. Under the cavitation effect of ultrasound treatment, the hydrophobic groups originally buried in the protein were exposed to the surface, which led to the enhancement of the hydrophobic interaction among molecules. [34]. Such migration behavior might be beneficial to the rapid formation of film structure after protein adsorption at the interface and increase the macroscopic foam capacity. Likewise, the corn starch granules further strengthened the hydrophobic interactions in the system, and this effect was positively correlated with the ultrasound intensity. Both surface hydrophobicity and secondary structure analysis results indicated that corn starch granules could exacerbate protein conformational changes on the basis of ultrasound modification. In this case, their interactions (hydrophobic interactions) were necessarily further enhanced. The amount of disulfide bonds in the WPI system was, moreover, significantly reduced after ultrasound intervention and was not affected by the corn starch granules. Disulfide bonds contributed a lot to the rigidity of protein molecules, so this phenomenon could be presumably inferred from the results of the analysis of molecular flexibility, even if the trends of them are not exactly the same. The disulfide bonds within and between the molecules of WPI were gradually disrupted under the interference of the local high-energy field caused by ultrasound. As well, this transformation actually provided favorable conditions for protein adsorption and rearrangement at the interface. The addition of starch granules did not lead to a further reduction of disulfide bonds, which indicated that starch granules would not affect the covalent interaction in the interface protein system. Recent research has proposed that interfacial film formed by too-soft proteins did not possess sufficient viscoelasticity to provide reliable mechanical resistance [27]. In this sense, CSG (20 mg/mL) played a key positive role in the stabilization phase of WPI foams, and the stability was improved by 41.40% under the optimal parameters.

## 4. Conclusions

Ultrasound treatment and CSG had significant positive effects on the foam capacity and stability of WPI, respectively. This phenomenon depends on suitable alterations in the molecular structure of WPI, and it is closely related to the Pickering stabilization mechanism of microgel granules (corn starch granules). Specifically, ultrasound treatment induces the unfolding of protein structure and a relative increase in flexibility through cavitation and local thermal effects. Furthermore, the inlay of corn starch granules at the Plateau interface prevented the intensification of the destabilization process which was achieved mainly by relying on the non-covalent interactions between WPI and CSG. This work will provide reliable and scientific insights for developing high-quality WPI foods and understanding the mechanisms of food protein foam evolution.

## Data Availability

The data presented in this study are available on request from the corresponding author.
